# Supplementary material for: The genome sequence of Sphagnum contortum Schultz, 1819 (Sphagnales: Sphagnaceae)
Source: Wellcome Open Res. 2026 May 29;11:312. [Version 1] doi: 10.12688/wellcomeopenres.26659.1 (PMC13550929; doi:10.12688/wellcomeopenres.26659.1)
Supplement: Peer review history

# Reviewer report: Reviewer response for version 1

**DOI:** 10.21956/wellcomeopenres.29359.r157652

**Published:** 2026-07-11

**Authors:** Jose Ramon Planta

**Affiliations:** 1 University of the Philippines Diliman, Quezon, Philippines

This manuscript reports the chromosome-scale assembly of the twisted bogmoss, *Sphagnum contortum*, as part of the Darwin Tree of Life project. Through PacBio and Hi-C sequencing, the twisted bogmoss genome is scaffolded into 21 pseudomolecules. 

 In the 'Methods -> Assembly curation' section, the curation process is described at https://gitlab.com/wtsi-grit/rapid-curation. This website, however, needs to be updated to reflect the current, actively maintained repository.

 There are 21 pseudomolecules described, although only 19 are shown in the contact map. Is it because Chromosomes 20 and 21 are too small to appear on the contact map? If so, please indicate in Fig. 3.

 Just to confirm, was the BUSCO score analysis performed in the genome mode? The chromosome-scale assembly for the twisted bogmoss has a low BUSCO score, which may reflect the genome's inherent properties.

Are sufficient details of methods and materials provided to allow replication by others?

Yes

Is the rationale for creating the dataset(s) clearly described?

Yes

Are the datasets clearly presented in a useable and accessible format?

Yes

Are the protocols appropriate and is the work technically sound?

Yes

Reviewer Expertise:

Plant molecular genetics and genomics

I confirm that I have read this submission and believe that I have an appropriate level of expertise to confirm that it is of an acceptable scientific standard.

# Reviewer report: Reviewer response for version 1

**DOI:** 10.21956/wellcomeopenres.29359.r156948

**Published:** 2026-06-19

**Authors:** Elisa S. Goldbecker

**Affiliations:** 1 University of Göttingen, Göttingen, Germany

The data note "The genome sequence of *Sphagnum contortum* Schultz, 1819" sequenced the genome of the twisted bog-moss *Sphagnum contortum* as part of the Darwin Tree of Life project. The genome was assembled using PacBio long-read sequencing as well as Hi-C data, yielding a high-quality, chromosome-scale assembly. Additionally, RNA was sequenced that will be used for annotation later on.

 The workflow is presented in a detailed and comprehensive manner, making use of state-of-the-art tools. The deposition of wet lab protocols in databases such as protocols.io is particularly useful. 

 Two minor comments: 

 The gitlab link: https://gitlab.com/wtsi-grit/rapid-curation should be replaced with the latest version of this repository https://github.com/sanger-tol/curation-resources. 

 It was not clear to me why the RNA extraction was listed here, when the samples were not used for downstream analysis. It would have been nice to present it together with the genome annotation.

Are sufficient details of methods and materials provided to allow replication by others?

Yes

Is the rationale for creating the dataset(s) clearly described?

Yes

Are the datasets clearly presented in a useable and accessible format?

Yes

Are the protocols appropriate and is the work technically sound?

Yes

Reviewer Expertise:

Evolutionary Biology, Molecular Biology, Bioinformatics

I confirm that I have read this submission and believe that I have an appropriate level of expertise to confirm that it is of an acceptable scientific standard.
